# Unmasking Racial Disparity in the Diagnosis and Treatment of Hidradenitis Suppurativa

**DOI:** 10.7759/cureus.41190

**Published:** 2023-06-30

**Authors:** Michelle R Anthony, Parsa Abdi, Christopher Farkouh, Howard I Maibach

**Affiliations:** 1 Pathology, University of Arizona College of Medicine, Tuscon, USA; 2 Faculty of Medicine, Memorial University of Newfoundland, St. John's, CAN; 3 Dermatology, Rush Medical College, Chicago, USA; 4 Dermatology, University of California San Francisco School of Medicine, San Francisco, USA

**Keywords:** black americans, minority representation, diagnosis delay, racial disparities, hidradenitis suppurativa

## Abstract

Hidradenitis suppurativa (HS) is a chronic, profoundly incapacitating disease predominantly affecting the apocrine gland-rich areas of the human body. Although it affects 0.05% to 4% of the general population, there exists a significant racial disparity, with people of color, particularly Black individuals, experiencing a notably higher prevalence. Despite this disparity, the current literature lacks comprehensive analyses of HS concerning race and ethnicity, revealing a systemic blind spot in understanding and addressing the disease's racially disproportionate impacts. In this commentary, we aim to shed light on these racial disparities, focusing specifically on the stark inequities related to the timely diagnosis and subsequent dermatological care of HS in the United States. This commentary explores the racial bias in HS prevalence, severity, diagnostic delay, access to specialized care, and underrepresentation in clinical trials. By emphasizing the urgent need to address these disparities, we seek to foster an inclusive dialogue and drive proactive efforts toward achieving equitable care and research representation for all populations affected by this debilitating condition. Through this discussion, we aim to pave the way for a healthcare landscape that acknowledges and addresses the racial disparities inherent in HS, ensuring that advancements in the management of the disease cater to the needs of all populations, irrespective of their racial or ethnic background.

## Editorial

Hidradenitis suppurativa (HS) is a debilitating and profoundly stigmatized chronic inflammatory cutaneous disorder that predominantly affects the apocrine gland-rich areas of the human body. The characteristic features of HS include painful and recurring nodules, abscesses, and sinus tracts located primarily in intertriginous areas, significantly impacting the quality of life of affected individuals [1,2].

While HS can affect individuals of any racial or ethnic background, there is a clear racial bias in its prevalence, with people of color, particularly Black Americans, experiencing a significantly higher burden of the disease. Garg et al. conducted a retrospective study that revealed a threefold higher prevalence of HS among Black Americans compared to their White counterparts [2]. Moreover, several independent studies have shown that African American and Hispanic American patients often exhibit a more severe manifestation of the disease, as indicated by elevated Hurley stage scores, compared to White patients [1].

The disproportionate burden of HS among people of color extends beyond prevalence and severity, with racial disparities observed in various aspects of the disease, including diagnostic delay and access to specialized care. Serrano et al. found that Black and Hispanic Americans experience a considerable delay in receiving a diagnosis, with an extended timeline of 1.6 and 1.5 years, respectively, compared to White patients [3]. This delay in diagnosis has profound implications for the management of HS, as it leads to a prolonged period of untreated disease and further exacerbation of symptoms.

Access to specialized dermatological care is crucial for effective HS management. However, there are significant inequities in accessing such care, particularly for Black Americans. On average, Black Americans consult with a dermatologist around five years after the onset of HS, which is approximately two years later than their White and Hispanic American counterparts [3]. This delay in accessing dermatological care potentially worsens the disease condition and may contribute to the increased need for surgical intervention in advanced HS cases. In fact, a staggering 44.9% of Black Americans with HS were evaluated by a surgeon before a dermatologist, potentially underlining the substantial barrier in accessing timely dermatological care within this population [3].

The racial disparities in HS management also extend to the realm of clinical trials, where the underrepresentation of minority populations further limits our understanding of the disease and its treatment efficacy. Price et al. conducted a study reviewing HS-specific clinical trials and found that out of 15 trials, only 14% of the included patients identified as Black Americans, while White Americans constituted a dominating 68% [4]. Similarly, Elhage et al. conducted a review and reported underrepresentation of Black, Hispanic or Latino, and American Indian or Alaska Native patients, constituting only 13.7%, 7.2%, and 1.3% of the trial population, respectively [5].

This significant disparity in representation not only limits our understanding of the disease but also perpetuates the racial disparities in HS management. Excluding diverse ethnic and racial groups from clinical trials undermines our ability to tailor treatments effectively and address the specific needs of these underserved populations. Inadequate representation in clinical trials may lead to insufficient evidence to support treatment decisions and may result in disparities in the availability and effectiveness of HS therapies for different racial and ethnic groups.

To address the racial disparities in HS diagnosis, treatment, and research, it is imperative for dermatologists and researchers to advocate for inclusivity within research studies. Efforts should be made to ensure the adequate representation of diverse racial and ethnic groups in clinical trials to improve our understanding of HS and develop more effective treatment strategies for all patients, regardless of their racial or ethnic background.

In addition to addressing representation in clinical trials, efforts should focus on raising awareness among healthcare providers about the racial disparities in HS. Education and training programs should emphasize the importance of culturally competent care and encourage dermatologists to consider the specific needs and challenges faced by people of color in HS management.

Furthermore, community outreach programs can play a significant role in addressing the disparities in HS care. By collaborating with community organizations, healthcare providers can raise awareness about HS, provide education on early detection and treatment options, and help individuals navigate the healthcare system to access appropriate care. These programs can also contribute to reducing the stigma associated with HS and promote a supportive environment for individuals living with the condition.

The management of HS requires a comprehensive approach that acknowledges and addresses the racial disparities inherent in the disease. By recognizing the higher prevalence, severity, delayed diagnosis, limited access to care, and underrepresentation in clinical trials experienced by people of color, particularly Black Americans, we can drive proactive efforts toward achieving equitable care and improving research representation for all populations affected by this debilitating condition. Addressing the racial disparities in HS requires a multifaceted approach involving inclusive research practices, education and awareness initiatives, and community outreach efforts. Only through these collective actions can we strive for a healthcare landscape that ensures equitable care for all individuals with HS, irrespective of their racial or ethnic background.
